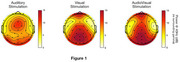# Spectris^TM^ combined audiovisual sensory stimulation elicits stronger EEG brain response than auditory or visual stimulation alone

**DOI:** 10.1002/alz70856_107772

**Published:** 2026-01-09

**Authors:** Chandran V Seshagiri, Brennan L Jackson, Miguel Hernandez, Julia M Leach, Ralph Kern, Alyssa Boasso, Mihaly Hajos

**Affiliations:** ^1^ Cognito Therapeutics, Cambridge, MA, USA

## Abstract

**Background:**

The recent Overture (NCT03556280) clinical trial demonstrated reduced decline in cognitive and functional abilities and reduced brain atrophy in participants with mild to moderate Alzheimer's disease (AD) after 6‐months of daily, at‐home treatment with Cognito Therapeutics’ Spectris™ investigational medical device (Hajós et al. 2024). Spectris uses auditory and visual sensory stimulation to evoke gamma‐frequency steady‐state oscillations. Here, we evaluate the acute EEG response to auditory and visual stimulation separately and compare with combined audiovisual stimulation.

**Method:**

All Overture participants underwent a screening EEG that included measurements of response to Spectris auditory, visual and audiovisual stimulation. Volume and brightness levels were matched for all stimulation within a subject. EEG was collected using gel caps (ANT‐Neuro, Philadelphia, PA) from 30 channels (10/20). EEG was preprocessed, visually inspected for a continuous 40s segment with minimal artifacts, and the power spectral density (PSD) was computed at each channel for each modality. The gamma response power was calculated as the ratio of the power at the stimulation frequency [40Hz] to the power in the surrounding gamma frequencies [32‐48Hz, exclusive of 39‐41Hz]. Statistics reported from ANOVA and Tukey's multiple comparisons test. Average global power reported as mean ± S.E.

**Result:**

Of 74 participants randomized, 41 participants had sufficient data quality from all 3 stimulation modalities for analysis. Figure 1 shows maps of the spatial distribution of the gamma response per modality. The number of channels with gamma response above a threshold of 6dB was greatest for audiovisual ‐ 24 vs 21 (auditory) or 22 (visual). The average global power across all electrode channels shows a significant effect of stimulation modality (*p* < 0.001). Audiovisual simulation [10.73 dB ± 0.56] was higher than auditory [7.39 dB ± 0.52, *p* < 0.001] and visual [10.01 dB ± 0.52, *p* = 0.49], but the difference was only significant between audiovisual and auditory. Similar results are seen when looking at Frontal or Occipital channels only.

**Conclusion:**

Spectris combined audiovisual stimulation produces the greatest spatial extent and the strongest average global gamma response. Further development and optimization of sensory stimulation therapy may maximize evoked brain responses through modality modifications.